# Determination of antimicrobial use in commercial poultry farms in Plateau and Oyo States, Nigeria

**DOI:** 10.1186/s13756-023-01235-x

**Published:** 2023-04-10

**Authors:** Mwapu Dika Ndahi, Rene Hendriksen, Birgitte Helwigh, Roderick M. Card, Idowu Oluwabunmi Fagbamila, Oluwadamilola Olawumi Abiodun-Adewusi, Eme Ekeng, Victoria Adetunji, Ini Adebiyi, Jens Kirk Andersen

**Affiliations:** 1 Department of Veterinary and Pest Control Services, Federal Ministry of Agriculture and Rural Development, Area 11, Garki, Abuja, Nigeria; 2 Technical University of Denmark, Kongens Lyngby, Copenhagen, Denmark; 3 Animal and Plant Health Agency, Weybridge, Woodham Lane, New Haw, Addlestone, Surrey, UK; 4 National Veterinary Research Institute, Vom, Plateau State Nigeria; 5 National Agency for Food and Drug Administration and Control, Lagos, Nigeria; 6 Nigeria Centre for Disease Control Laboratory, Gaduwa, Abuja, Nigeria; 7 University of Ibadan, Ibadan, Nigeria; 8 University College Hospital, Ibadan, Nigeria

**Keywords:** Antimicrobials, Antimicrobial resistance, Antimicrobial use, Active ingredients, Poultry and kobotool

## Abstract

**Background:**

Indiscriminate use of antimicrobials for the prevention and treatment of bacterial infection in animals is a common practice in Nigeria as in other developing countries. These antimicrobials are purchased over the counter without restrictions and often administered in form of medicated feedstuffs. In Nigeria, like most developing countries, antimicrobial prescription data are not routinely collected or reported at the farm level, instead import data are used in reporting antimicrobial consumption. Farmers can be useful sources of data on the use of antimicrobial agents by class, animal species, production type and age. The objective of the study was to determine the knowledge, attitude and practices of poultry farmers on antimicrobial resistance and to generate data on antimicrobial use (AMU) in poultry farms in Plateau and Oyo states in accordance with the guidelines of the World Organization for Animal Health (WOAH).

**Methods:**

A questionnaire used by the Food and Agriculture Organization (FAO) of the United Nations in Ghana was adopted and modified to collect data on the knowledge, attitude and practices of farmers on AMR and AMU and to collect AMU data from selected poultry farms. A focus group discussion (FGD) was conducted in Plateau state with poultry farmers and representatives from the state veterinary services, using a checklist. The aim of the FGD was to have an idea on antimicrobial use among poultry farmers and to generate additional questions that might be added to the questionnaire. Stratified random sampling technique was used to select 50 farms from Plateau and Oyo states, using the list of registered poultry farms in the two states as sampling frame.

**Results:**

Ninety eight percent (98%) of farmers gave antibiotics as prophylactic treatment to day old chicks. There were 47 different products used in the two states within the study period. We observed that five classes of antibiotics (Tetracyclines, Penicillins, Aminoglycosides, Polypeptides and Fluoroquinolone) were used in the two states. A total of 351 kg of active ingredients from seven different classes, namely: tetracyclines, penicillins, aminoglycosides, polypeptide, fluoroquinolones, amphenicol and macrolides were recorded from the two states. Some products contained cocktail of antibiotics, having up to six different classes with very high concentration of active ingredients which are not in the list of registered antimicrobials reported to WOAH.

**Conclusion:**

The concept used for this survey proved that the approach can be applied for AMU surveillance in the animal health sector. It also provided insight on farmers’ knowledge and practices with regards to the use of antimicrobials which is missing in the national import data. The need for “stronger” antibiotics was identified as one of the drivers of antibiotic resistance.

**Supplementary Information:**

The online version contains supplementary material available at 10.1186/s13756-023-01235-x.

## Background

Nigeria is a Federation of 36 States and the Federal Capital Territory with a population of about 200 million people and a livestock population of about 349 million animals out of which 194 million are poultry [1].

Antimicrobial agents are used in livestock production to ensure good health and productivity of animals [2]. However, the inappropriate use of these drugs especially when the classes are the same as, or related to, the pharmaceuticals used in the control of human infections [3] in the livestock sector, or the use of substandard/sub-optimal dose may predispose to antimicrobial resistance (AMR). Antimicrobial resistance is an emerging One Health issue that can be transmitted between animals, humans and the environment and is able to spread across the globe. The global antimicrobial usage in food animals was estimated at 63,000 tons annually in 2015 and projected to increase by almost 70% in 2030 [2]. However, this may change due to the increased awareness of the problem.

The surveillance of antimicrobial use in animals is more complex than in humans due to the variation in use patterns by different animal species and production types (e.g., beef and dairy cattle). In order to monitor antimicrobial use, the WOAH developed standards on “Monitoring of the quantities and usage patterns of antimicrobial agents used in food producing animals” [4]. Nigeria has been submitting data on the amount of active ingredients of antimicrobial agents intended for use in animal health using import data. The data on antimicrobial agents for use in animals were obtained from the National Agency for Food and Drug Administration and Control (NAFDAC). The amount of active ingredients in each antimicrobial was calculated and converted into kilograms as described in the WOAH guidance] for reporting antimicrobial agents (AMUse guidance – Additional file 1).

The amount of active ingredients of antimicrobial agents were 207; 516; 331 and 339 thousand kilograms for the years 2014 to 2017 respectively, although the data for 2014 was for 6 months only. The classes of antimicrobial agents include Tetracyclines, Fluoroquinolones, Macrolides, Penicillin, Sulfonamides, Polypeptides, Aminoglycosides, Amphenicols, Glycopetides, Pleuromutilins and Nitrofurantoin (Antimicrobial Use Report-Federal Department of Veterinary and Pest Control Services, Nigeria). Due to its carcinogenic effect, Nitrofuran was in 2019, banned for use in livestock feed in many countries, including Nigeria [5].

These antimicrobials are purchased over the counter without restrictions and in livestock management, the use of antimicrobials for therapeutic and prophylactic purposes is common which is often administered in form of medicated feedstuffs [6].

There is evidence of overreliance on and indiscriminate use of antimicrobial drugs among broiler farmers across six stages of the value chain in Oyo state, Nigeria [7]. The study also revealed that 80% of the farmers interviewed utilized antimicrobial drugs as preventive or therapeutic drugs without laboratory diagnoses and veterinary prescriptions. Furthermore, most of the farmers indicated non-compliance to withdrawal period. Non-therapeutic antimicrobial use, particularly for growth promotion or prophylaxis, has generated significant concern due to increasing evidence of its contribution to AMR [8]. NmaBida and Tajudeen [9] found out that 58.3% of herders in North central Nigeria practiced self-prescription for their livestock and 23.2% use antimicrobial agents as growth promoters. Adesokan et al. [3] reported an increase in antibiotic consumption in animal health in Southwestern Nigeria between 2010 and 2012 and the study revealed tetracyclines, fluoroquinolones and betalactams/aminoglycosides as the leading antimicrobials used in livestock production.

Studies carried out in Nigeria in cattle, poultry, pig, goat, vegetables, human, bats, camel, sheep, and fish showed residues of antimicrobial agents in animals, as well as the presence of multiple drug resistant isolates [7, 10]. Other studies showed the presence of multiple drug resistant bacteria in meat and ready to eat meat products in Nigeria [11]. It is a well-known fact that drug-resistant pathogens are currently responsible for about 700 000 deaths annually and this is likely to increase to 10 million by 2050 if left unchecked, which is expected to affect global economy [12].

In Nigeria, like most developing countries, antimicrobial prescription data are not routinely collected or reported from the farm level, instead import data is used in reporting antimicrobial use. Farmers can be useful sources of data on consumption of antimicrobial agents by class, animal species, production type and age. In some countries, farmers are required to maintain records of treatment, which can be a valuable source of data. In Nigeria, however, this is not the case. Therefore, it may be necessary to carry out data collection on a subset or a sample of farms.

## Methodology

### Study design, site and method

The study was a cross sectional study aimed at establishing a proof of concept for antimicrobial use data collection at farms level.

The study was carried out in Plateau and Oyo states in Nigeria. Plateau State is in the middle belt of Nigeria with 17 Local Government Areas and a population of about 3.5 million people [13]. It has a near temperate climate with an average temperature of between 13 and 22 °C. Poultry population in Plateau State is estimated at 8 million [1]. Poultry farming thrives very well in Plateau State because of the relatively cool weather and poultry eggs tend to have longer shelf life (conversation with poultry farmers and marketers). Oyo State is in the South-western Nigeria with a population of about 6 million people [14]. The Climate is characterized by dry and wet seasons with relatively high humidity and average temperatures between 25 °C and 35 °C, almost throughout the year. The poultry population in Oyo State is estimated at 12 million [1].

The reasons for selecting these states are to have a representation from the Northern and Southern parts of the country and the fact that both states have relatively high poultry population [1] as well as easy accessibility of data from poultry farms. Furthermore, the two states have different weather conditions which makes it appropriate for comparison and finally, availability of contacts of the federal and state officers and those of the poultry farmers.

The aim of the study was to determine the level of antimicrobial resistance (AMR) awareness among poultry farmers; develop an effective system for AMU surveillance and to generate data on AMU in selected poultry farms in Plateau and Oyo States, Nigeria.

The specific objectives of the study were to determine the knowledge, attitude and practices of poultry farmers with regards to antimicrobial use through a structured questionnaire administration, to find out if farmers practiced biosecurity and vaccination as disease preventive measures through questionnaire administration; and to identify the types and quantity of antimicrobial agents prescribed/used in poultry through questionnaire administration and provision of evidence;A questionnaire used by the Food and Agriculture Organization (FAO) of the United Nations [15] in Ghana was adopted and modified to collect data on the knowledge, attitude and practices of farmers with regards to antimicrobial use and to also collect AMU data from selected poultry farms.

#### Focus Group Discussion

A focus group discussion (FGD) was conducted in Plateau state with officials of poultry farmers association and representatives from the state veterinary services, using a checklist. The purpose of the focus group discussion (FGD) was to obtain additional information on antimicrobial use among poultry farmers to generate additional questions that might be added to the questionnaire. Questions in the checklist for the FGD included poultry production type in the state, poultry management system, common poultry diseases, access to veterinary services, access to antimicrobials and frequency of use, repeat treatments and what farmers do when birds do not recover from treatment with antimicrobials. The responses of farmers and veterinary officials from the FGD provided more insight on poultry production system and farmers practices, which were used to fine-tune the questionnaire.

#### Use of Kobo tool for imputing questions

The draft questionnaire was imputed into Kobo toolbox (https://www.kobotoolbox.org) and deployed onto android phone for ease of administration. A link was created to provide access to the questionnaire for farmers. The idea was for farmers to be able to fill out the questionnaire on their android phones. All questions related to farmers’ knowledge, attitude and practice on AMR as well as questions to obtain quantity of antimicrobial agents used are found in the following link (https://ee.humanitarianresponse.info/single/gOqBgWQO).

### Pretest of questionnaire

Twelve farmers were identified by the farmers association for the pretest. The questionnaire was administered physically to seven farmers, while five farmers were sent the link to fill out the questionnaire.

Information was only obtained from farmers to which questionnaire was administered physically. The farmers that got the link to the questionnaire made no submission.

The result of the pre-test was used to finalize the questionnaire.

Convenience sampling method was used to select 50 poultry farms from Plateau and Oyo states.

Stratified random sampling technique was used to select 25 farms each from Plateau and Oyo states, using the list of registered poultry farms in the two states as sampling frame. Therefore, a total of 50 farmers were interviewed.

### Farmers’ interview using questionnaire

All farmers that were contacted gave their consent to respond to the questionnaires.

Interviews were conducted for 50 poultry farmers during a three month study period using Questionnaires on kobo collect tool on android phone and completed forms were uploaded (submitted) to the Kobo toolbox on the computer. Data collected include farmers knowledge, attitude and practices on antimicrobial resistance and use; use of biosecurity and vaccination as means of reducing the impact of antimicrobial resistance and data on antimicrobial use in poultry.Survey data were downloaded from Kobo toolbox to Microsoft excel. Data collected during the pre-test were excluded from the final analysis.

Data on antimicrobial agents used in poultry was for a period of three months during the study period. The amount of active ingredients in each antimicrobial agent was calculated and converted into kilograms as described in the WOAH guideline for reporting antimicrobial agents (Additional file 1).. All antimicrobial agents were grouped into the various classes. Antimicrobial agents having more than one active ingredient were disaggregated into the respective classes and amount of active ingredients calculated. Microsoft excel was used in analyzing data. Results were presented in tables, percentages, graphs and charts.

## Results

### Farmers’ demographic information and production system

In the two states, there were more males (80%) involved in poultry farming than females (20%) and the majority (58%) of the farmers were above 50 years of age. There seemed to be a tendency that retired civil servants invest their retirement benefits in poultry farming as a means of sustaining their families, and as a result, 68% of the farmers were into full time poultry farming. Only 4% were below the age of 40. All the farmers had some level of formal education; 58% had tertiary education, 30% had postgraduate education and 12% had secondary school certificate. It is mandatory for poultry farmers to be registered with the government in some states, including Plateau and Oyo. The study showed that 90% of the farmers were registered with the government and poultry farming is the main occupation of majority (78%) of the farmers in both states.

The farms were on the average, between 6 to 8 years of production with most (44%) farms having three pens per farm with an average of 1000 birds per pen in the two states. Sixty percent (60%) of farmers had layers belonging to sector 3 poultry production system according to FAO classification [16]. Although all the farmers started with day-old-chicks, at the time of the study, 78% of the farmers had laying hens above 23 weeks of age in both states. The predominant breed of poultry were Isa Brown and Lohman Brown. All farmers kept age groups in separate pens and sold poultry manure to crop and vegetable farmers.

### Poultry disease information and use of antimicrobials

**Fig. 1:**
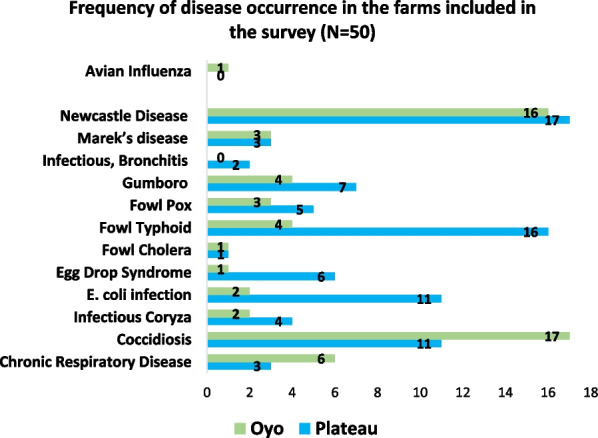
Frequency of disease occurrence in the farms included in the survey

The administration of antibiotics as prophylactic treatment to day old chicks was exceeding common amongst all farmers (98%). All farmers reported issues with diseases on their farms and the most common diseases reported were:: Newcastle Disease Chronic, Coccidiosis, Fowl Typhoid, *Escherichia Coli* (*E.coli*) infection, Chronic Respiratory Disease, Egg Drop Syndrome, Fowl Pox, Gumboro, Marek’s disease, Infectious Coryza, Infectious Bronchitis, Fowl Cholera and Avian Influenza (Fig. 1).

Newcastle was the highest recurring disease in poultry farms in the two states, followed by Coccidiosis, Fowl typhoid and *E. coli* infection. Coccidiosis was higher in Oyo state and this could be due to the fact that Oyo state has a warmer temperature and higher humidity than Plateau state. Fowl typhoid and *E. coli* infection were higher in Plateau state, although the reason for that was not clear except for the fact that most farmers used well water in Plateau state as against deep borehole water in Oyo state.

### Farmers’ knowledge on antimicrobial resistance

Seventy percent (70%) of farmers have heard about antimicrobial resistance; 29% said they read about it; 59% heard about it from private/government veterinarians; and 12% from a family member. Majority of the farmers (92%) thought antimicrobial resistance would have great impact on them, their families/friends and their animals. All farmers agreed that it was important to get consultation from a veterinarian before giving antibiotics to poultry.

Most farmers (70%) did not know about antimicrobial residues, however, 74% read about withdrawal period from the antibiotic containers/sachets, yet all farmers interviewed sold eggs under current treatment with antibiotics. Since there was no provision for compensation by government, the farmers stated that the eggs were sold.

**Fig. 2:**
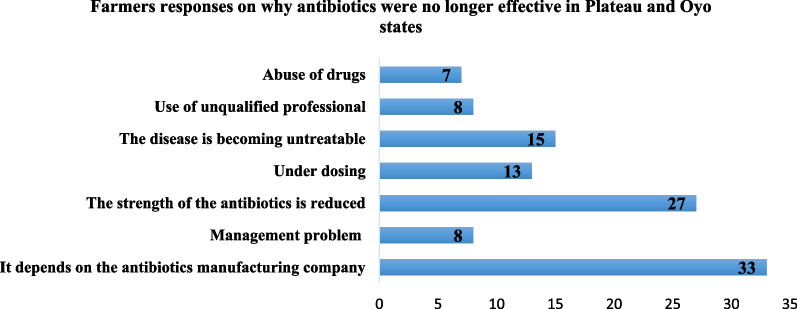
Farmers responses on why antibiotics were no longer effective

Most farmers thought that antibiotics were no longer effective because of the manufacturing company; reduced strength of the antibiotics and the fact that diseases were becoming untreatable (Fig. 2). Although farmers interviewed in this survey have heard about AMR, this highlights the lowperception of AMR among farmers and the need for awareness creation and sensitization.

### Farmers’ attitude and practices towards antimicrobial use

All farmers in the two states bought and used antibiotics to treat infections. Although most farmers (84%) consulted private or government veterinarians when the birds were sick, 16% bought antibiotics from the drug store without consulting veterinarians, reason being that they consider to have been in the poultry business long enough to know what antibiotics to give when the birds were sick. Farmers in Nigeria have been reported to treat animals with antimicrobials without prescription [9]. Seventy-four percent (74%) of farmers bought antibiotics from poultry drug stores, while the rest (26%) obtained theirs from private and government vets. Fifty eight percent (58%) of famers covered a distance of 3 to 5 kms to buy antibiotics in the two states. Therefore, antimicrobial agents were readily accessible to farmers.

**Fig. 3:**
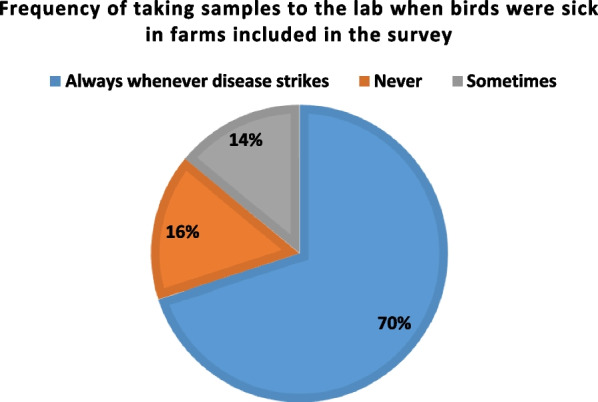
Frequency of taking samples to the lab when birds were sick in farms included in the survey

Seventy-two percent (70%) of farmers took samples to the lab always; 14% took samples to the lab sometimes, while 16% never took samples to the lab (Fig. 3).

The challenges associated with taking samples to the lab were described by the farmers as far distance to the lab (36%) and high cost of analysis (30%) (Table 1).

**Table 1:** Farmers’ attitude to antimicrobial use

Farmers’ attitude to antimicrobial use	Agree (%)	Disagree (%)	Indifferent (%)
Farmers observe withdrawal period before selling or eating animal products	74	16	10
Farmers stop giving antibiotics if symptoms improve	30	70	0
Farmers believe that if antibiotics are given too often, they might stop working	90	8	2
Farmers believe that giving animals that are not sick antibiotics will prevent them from becoming sick in the future	60	34	6
Farmers believe that giving animals antibiotics can help them grow bigger, faster, fatter	32	64	4

When farmers observed that the antibiotics they used regularly were no longer effective, 62% consulted animal health professionals; 24% tried different antibiotics; while 14% increased the dosage of antibiotics. When birds were sick, most farmers separated sick birds from healthy ones but the entire flock undergoes treatment.

Ninety-eight percent (98%) of farmers gave antibiotics as prophylactic treatment to day old chicks.

### Types of antimicrobials used and amount of active ingredients in kilograms

In Plateau state, a total of 39 different antibiotics products from six different classes were used for treating poultry during the three month study period (Additional file 2). Within that period, 88% of the farmers interviewed used 311 kg of active ingredients of antibiotics to treat poultry (Table 2). In Oyo state, a total of 24 different antibiotics products from six different classes were used for treating poultry during the three month study period (Additional file 3). Most of the farmers interviewed in both states administered antibiotics to poultry on a monthly basis, as prophylaxes; just before the “monthly” vaccination against Newcastle disease. All farmers interviewed in Oyo state used 40 kg of active ingredients of antibiotics to treat poultry within the three month study period (Table 2).

**Table 2:** Amount of active ingredients of antibiotics used within three months in poultry farms included in the survey

Antibiotics	Amount of active ingredients in kg (Plateau)	Amount of active ingredients in kg (Oyo)
Tetracycline	290	15.4
Penicillin	2	0.2
Aminoglycoside	4	7.4
Polypeptide	12	0.1
Fluoroquinolone	2	3.8
Amphenicol	1	0
Macrolide	0	13.1
Total	311	40

All antibiotics except the human preparation of gentamycin were given in water to layers of all age groups as treatment (55%) and prophylaxes (45%); 84% of the birds were actively laying during the period of the study.

Of concern was the fact that some products contained cocktail of antibiotics, having up to six different classes with very high concentration of active ingredients e.g.,

- Neo-furamycin® (furazolidone 6000 mg, neomycin 200 mg, oxytetracycline 500 mg, streptomycin 200 mg, erythromycin 3500 mg, chloramphenicol 2000 mg);
- Embaceryl® (tylosin base 3,800 mg, oxytetracycline 4,000 mg, neomycin sulphate 1,200 mg, colistin sulphate 30,000,000 IU).

Others have 3 molecules in one e.g.,

- Neo-furaseryl® (neomycin 100 mg, oxytetracycline 50,000 mg, colistin sulphate 30,000,000 IU plus vitamins);
- Zogceryl^®^ (oxytetracycline 5,000 mg, colistin 2,500 mg, neomycin 200 mg);
- Maxiceryl**®** (colistin 225000 IU, oxytetracycline 5000 mg, neomycin 5000 mg);
- Keproceryl® (oxytetracycline 50 mg, colistin sulphate 225,000 IU and erythromycin 35 mg) and,
- N.C.O mix® (florfenicol 150 mg, neomycin sulphate 180 mg, colistin sulphate 1,200,000 IU).

The need for antibiotics with multiple molecules and high concentration of active ingredients to treat infections in poultry by the farmers interviewed could be a potential driver of antibiotic resistance.

Furazolidone (Oxazolidines) were being used by some farmers in poultry in combination with other antibiotics even though it was banned for use in food producing animals by the regulatory authorities in Nigeria. Amphenicol was found to be used by few farmers even though it is not in the national import data reported to the WOAH for the past four years. We also observed that some products with very high concentration of active ingredients such as Neo-furaseryl®, Floricol and Furamycin were not reported in the national import data, which is an indicator for importation through other means.

Some farmers used products containing a mixture of antibiotics and probiotics such as Biodox® (Doxycycline and Lactobacillus). Fifty-six percent (56%) of farmers interviewed used human preparation of injectable gentamycin (80 mg of 2 ml vials); and Septrin® (Sulphur/Trimethoprim) in water because the birds were not responding to treatment. Others used *Zingiber officinale *(ginger) in water as prophylaxes.

Some farmers administered disinfectants in drinking water to poultry (e.g., Viru supa disinfectants®, containing potassium peroxomonosulfate 59% and sodium dichloro-iso-synurate 10%; Corygiene®); and antiseptics such as Fivevet® (iodine preparation) and Aquaseptic®) in drinking water.

Farmers tend not to buy more antibiotics than they need at a time, but in case antibiotics expire, 96% said they would throw them away. In case of mortalities, 56% of farmers fed dogs with the carcasses.

We observed that five classes of antibiotics (Tetracycline, Penicillin, Aminoglycoside, Polypeptide and Fluoroquinolone) were consistent in the two states. This is in agreement with the national AMC data submitted to the WOAH for the past six years [17]. However, amphenicol was only reported in Plateau state and likewise macrolide was only reported in Oyo state. The reason for the variation is not clear. Also, the amount of tetracycline was higher in Plateau state and this might be attrbuted to the type of products (products with very high concentration of active ingredients) used by poultry farmers.

### Information on vaccination and biosecurity

All farmers interviewed began with day-old-chicks as starter flock and 72% stated that the chicks were vaccinated at day old against diseases such as Marek’s from the hatcheries. All farmers vaccinated poultry against the following diseases: Newcastle disease, Gumboro disease, Coccidiosis, Fowl pox, Fowl typhoid, Marek’s disease, Egg drop syndrome, Fowl cholera and Avian encephalitis. Although there is a “No vaccination” policy on Avian Influenza in Nigeria, we found out during the course of the study that some farmers vaccinated against the disease. Following vaccination, some birds still came down with the same disease in some of the farms. Fifty-two percent (52%) of farmers agreed that using vaccines could prevent the use of antibiotics, while 34% disagreed and 14% were indifferent.

Fifty-eight percent (58%) of farms included in this survey were fenced to regulate human and animal traffic as a form of biosecurity. All the pens had wire mesh and the floors made of concrete for ease of cleaning and disinfection. All the farms had foot dip at the entrance, and most farmers (56%) changed the disinfectant water on a daily bases, while others changed on a weekly (22%) or monthly (22%) bases. Ninety-two percent (92%) of the farms had changing rooms and workers wore specific clothing and boots during work on the farm; 96% of the workers were almost always permanently assigned to work in separate pens. None of the staff worked on other farms and only 20% had poultry at home. All farmers cleaned and disinfected pens after selling each batch of poultry.

Most farms (96%) were neighbors with other poultry farms within 1 km radius and almost all farmers allowed their farms to rest for at least one month before restocking.

Although a greater percentage of the farms were fenced, 64% of customers came into the farms to buy eggs, which is a risk factor for disease introduction. A risk assessment carried out by the Federal Department of Veterinary and Pest Control Services during the outbreak of Avian influenza in Nigeria in 2016, identified eggs and manure merchants as sources of transmission of the pathogen from infected to non-infected farms through used paper crates and used sacks.

Even though most farmers practiced good biosecurity, all the farms visited had disease problems.

Ninety-six percent (96%) of farmers kept farm records including sales/financial, mortality, vaccination and medicine records.

The fact that 72% of farmers bought commercial feed was advantageous in reducing the spread of disease-causing pathogens that could occur through exchange of sacks at toll milling stations. Although 92% of farmers admit that additives were added to the feed they used for poultry, none contained antibiotics.

All farmers used well or borehole water for poultry and drinkers were washed on a daily bases by most farmers.

## Discussion

The use of structured questionnaire enabled us to collect actual AMU data at the farm level. Seven classes of antimicrobial agents (Tetracycline, Penicillin, Aminoglycoside, Macrolide, Fluoroquinolone, Amphenicol and Polypeptide) were identified in the two states during the three month study period. Tetracyclines accounted for the highest antibiotics used in both states, followed by Polypeptides, Aminoglycosides and Fluoroquinolones respectively. This finding is in concordance with the annual AMC data reported to the WOAH by Nigeria [Federal Department of Veterinary and Pest Control Services (FDVPCS)]. Adesokan et al. [3] also reported tetracyclines, fluoroquinolones, beta lactams and aminoglycosides as the leading antimicrobials used in livestock production in Nigeria. A total of 351 kg of active ingredients of antibiotics were used in poultry in the farms visited within the three month study period.

The study revealed that farmers used cocktail of antibiotics, having up to six different classes with very high concentration of active ingredients. The study also revealed that farmers were using antibiotics banned by the regulatory authorities even though such products are not captured in the annual import data reported to the WOAH. These practices could aggravate the development and spread of resistance by microorganisms through selective pressure.

Farmers interviewed used antibiotics to treat infections, though not always based on laboratory test and prescription from veterinarians. The continuous overuse of antimicrobial agents by poultry farmers in this study could pose a public health threat, thereby exposing humans to subclinical doses of antimicrobials through the food chain, especially since some antimicrobials are potentially carcinogenic, allergenic mutagenic and teratogenic. Some farmers have resolved to using human preparations of antibiotics especially injecting birds with gentamycin for treating infections that did not respond to treatment.

Most farmers interviewed were retirees, above the age of 50 who have used their retirement benefits to start up poultry farming as a main source of livelihood. Most of the farms belonged to sector 3 as categorized by the FAO [16]. Although all farms had some form of biosecurity measures in place, rationale behind the measures was not fully understood by farmers, for example, farmers allowed eggs and manure merchants into the farms. Hence, all farms visited were faced with disease problems in poultry such as Newcastle Disease, Coccidiosis, Fowl Typhoid, *E. coli* infection, among others. Newcastle disease was the highest most recurring disease in the two states. Coccidiosis was found to be higher in Oyo state.

Although most farmers interviewed have heard of antimicrobial resistance, there was a low-risk perception of antimicrobial resistance among them. The repeated use of antimicrobials as prophylaxis instead of applying preventive measures such as improved management practices and biosecurity measures is an indication of low-risk perception. Hence the need for continuous awareness creation and sensitization of farmers on the danger of AMR and trainings on the use of alternatives to antimicrobials such as improved biosecurity practices, effective vaccination and the use of pre and probiotics. The belief that antibiotics were no longer effective because of reduced strength or because diseases were becoming untreatable and not due to overuse of antibiotics or poor management practices further buttressed this point. This study revealed the possibility that a majority of eggs in the market might contain some residues of antimicrobial agents since eggs were sold during treatment. This would warrant further study and efforts to improve farmer practices to minimize any risk to the food chain.

Despite the fact that all farmers vaccinate birds against preventable diseases and observe some level of biosecurity measures, the fact that customers came into the farms to buy eggs were identified as high-risk factors for disease entry into farms and resultant increase in the use of antibiotics.

## Conclusion

The use of structured questionnaire for this survey proved that the approach can be applied for AMU surveillance in the animal health sector. It also provided some insight on farmers’ practices with regards to the use of antimicrobials which is missing in the national import data. It can therefore be adopted to improve AMU surveillance in Nigeria. The concept is already being further developed and expanded for national AMU data collection in all the states of the federation, pending availability of funds.

There is also clear evidence that the current surveillance of AMU using the national import data is missing some important data. Especially the fact that antimicrobials such as Furazolidone is being used by some farmers in poultry in combination with other antibiotics even though it was banned for use in food producing animals by the regulatory authorities in Nigeria. Also, the fact that Amphenicol was found to be used by few farmers on the field even though it is not in the national import data reported to the WOAH for the past four years. We also observed that some products with very high concentration of active ingredients such as Neo-furaseryl®, Floricol and Furamycin were not reported in the national import data, which is an indicator for importation through other means. The use of human preparations of Septrin® and Gentamycin injection in poultry to treat resistant strains is a clear indication of resistance to antibiotics used in poultry. Five classes of antibiotics (Tetracycline, Penicillin, Aminoglycoside, Polypeptide and Fluoroquinolone) were used in the two states, however, amphenicol was only reported in Plateau state and likewise macrolide was only reported in Oyo state. This study is therefore a pointer that the approach used could be applied to gather useful information directly from the farms as well as generate and calculate the actual amount of antimicrobial agents used in poultry using the WOAH guidelines. The fact that AMU data was generated during the survey indicated the possibility of using this approach for national AMU surveillance.

In order to generate the actual antimicrobial use data, there is need to collect data from the farms and clinics levels especially since some antibiotics reported by farmers are not captured in the import data. There is also the need to increase awareness among poultry farmers on the importance of biosecurity; disease preventive measures such as vaccination and promote the use of probiotics to enhance production. These would reduce disease introduction to farms and ultimately reduce the use of antibiotics. Increased awareness creation and sensitization among private veterinarians and poultry drug sellers on the danger of AMR and the need for prescriptions from laboratory test results before prescribing or selling antibiotics to farmers will go a long way in mitigating AMR.

## Supplementary Information

**Additional file 1.** Annex to the Guidance for Completing the OIE template for the collection of data on Antimicrobial Agents intended for use in Animals.

**Additional file 2.** List of antibiotics used on farms (Plateau).

**Additional file 3.** List of antibiotics used on farms (Oyo state).

## Data Availability

A link to the questionnaire is given below: https://ee.humanitarianresponse.info/single/gOqBgWQO
