# Supplementary material for: Determination of antimicrobial use in commercial poultry farms in Plateau and Oyo States, Nigeria
Source: Antimicrob Resist Infect Control. 2023 Apr 10;12:30. doi: 10.1186/s13756-023-01235-x (PMC10084607; doi:10.1186/s13756-023-01235-x)
Supplement: Supplementary file 1 — Additional file 1. Annex to the Guidance for Completing the OIE template for the collection of data on Antimicrobial Agents intended for use in Animals. [file 13756_2023_1235_MOESM1_ESM.pdf]

Annex to the Guidance for Completing the OIE template for the collection of data on Antimicrobial Agents intended for use in Animals:

Considerations on converting content of antimicrobial active ingredients in veterinary medicines into kilograms

Calculating the quantities to report in kilogram (kg)

Data on antimicrobial agents intended for use in animals comes in different forms. The OIE template for the collection of data on antimicrobial agents used in animals (OIE template) is designed to collect data on the amounts of chemical compound as declared on the product label. The information may vary, ranging from bulk quantities of antimicrobial agents to numbers of packs of a veterinary medicinal product. The content of antimicrobial agents in such products can be stated in a number of possible ways. It will be necessary, where appropriate, to calculate the required data to populate the OIE template.

Detailed instructions are provided to harmonise some aspects of data reporting:

- Transformation of bulk quantities (section 1);
  use this section if you need to convert quantities of raw material, e.g. from import data into the required format.
- Data on veterinary medicinal products (section 2), including conversion from International Units (IU) to kg (section 2. (ii))
- Recommendations are made in section 3 for further optional conversions, aimed at achieving refined reporting of active entities, the ultimately desired format. If such calculations are made, they should be reported in the OIE template in the free text field provided on the sheets for Reporting Option 1, 2 and 3.

The following abbreviations and symbols will be used:

Symbol/abbreviation	Explanation
Strength	amount of antimicrobial agent per unit of veterinary product
% w/v	per cent weight per volume
mg	milligram
g	gram
kg	kilogram
t	ton (metric)
ml	millilitre
l	litre

1. For data on bulk quantities

Such information is usually sourced from customs, import or other bulk trading. It will likely come as a weight in a number of possible units (e.g. metric tons) of chemical compound and needs to be converted to kg. When conversion into kg is necessary, follow the steps below. If additional conversion factors are needed, please contact the OIE at antimicrobialuse@oie.int.

Step 1: Multiply the amount of antimicrobial agent, i.e. the chemical compound as declared on the product label with the appropriate conversion factor from the table 1 below.

$$Antimicrobial\ agent\ (kg) = antimicrobial\ agent\ (unit\ Z)\ x\ conversion\ factor$$

Table 1: Converting weight units into kg

Unit reported (unit Z)	Conversion factor to kg (for multiplication)
Metric ton	1000
Imperial ton (long)	1016
Imperial ton (short)	907.18
Stone (Imperial)	6.35
Imperial Pound	0.4536
Ounce	0.0283

2. For data on veterinary medicinal products

For veterinary medicinal products containing antimicrobial agents, data on quantities sold is likely to be available as numbers of packages of product sold, with each package containing a specified quantity of medicinal product with a specified amount of antimicrobial agent. In such cases, the amount of antimicrobial agent (chemical compound as declared on the product label) per package needs to be calculated first, and subsequently the result needs to be multiplied with the number of packages of the presentation sold to obtain the overall amount of antimicrobial agent, which should be reported in kg.

The most common ways to indicate the content of the antimicrobial agent(s) of a veterinary medicinal product are:

(i) Strength in mg or g of the active ingredient per volume or weight or other unit, (for example: ml, l, kg, tablet),
(ii) Strength in International Units (IU) per weight, volume or other unit,
(iii) Strength in per cent (%) weight per weight (w/w) or weight per volume (w/v).

Each situation requires a different kind of mathematical conversion.

2. (i) – content of antimicrobial active ingredient (antimicrobial agent) stated in milligram per volume or weight or other unit (for example millilitre, litre, kilogram, tablet) of content

Step 1: Calculation of the content of antimicrobial agent per package

Multiply the amount of antimicrobial agent (chemical compound as declared on the product label) per unit of content, that is, the strength of the product, with the total number of units contained in the package

$$Content\ of\ antimicrobial\ agent\ per\ package$$
$$= Strength\ (amount\ antimicrobial\ agent\ per\ unit) x\ number\ of\ units\ per\ package$$

Example A:
Tiamulin 100 g/kg premix for medicated feeding stuff; package sizes: (a) 1 kg, (b) 5 kg and (c) 20 kg

Calculation of content of antimicrobial agent, tiamulin, per package:

(a) $Pack\ content = 100\ g/kg\ x\ \ 1\ kg = \ 100\ g$
(b) $Pack\ content = 100\ g/kg\ x\ \ 5\ kg = \ 500\ g$
(c) $Pack\ content = 100\ g/kg\ x\ 20\ kg = 2000\ g$

Example B:
Tetracycline intrauterine tablet containing 2000 mg tetracycline hydrochloride per tablet; package sizes: (a) carton with 1 blister of 5 intrauterine tablets, (b) carton with 4 blisters of 5 intrauterine tablets each (20 tablets), (c) carton with 20 blisters of 5 intrauterine tablets each (100 tablets).

Calculation of content of antimicrobial agent, tetracycline, per package:

$(a)\ Pack\ content = 2000\ mg\ \ x\ \ 5\ = 2\ g\ x\ \ 5\ = \ 10\ \ g$
$(b)\ Pack\ content = 2000\ mg\ \ x\ \ 20\ = 2\ g\ x\ \ 20\ = \ 40\ \ g$
$(c)\ Pack\ content = 2000\ mg\ \ x\ 100\ = 2\ g\ x\ 100\ = 200\ \ g$

Example C:
Tilmicosin 300 mg/ml solution for injection for cattle; package sizes: containers of 100 ml and 250 ml; packs of *(a)* 6, *(b)* 10 and *(c)* 12 units of 100 ml and 250 ml.

Calculation of content of antimicrobial agent, tilmicosin, per package:

(a) Container content = 300 mg/ml x 100 ml = 30000 mg = 30 g

Pack content: *(a) 6 x 30 g = 180 g,*
(b) 10 x 30 g = 300 g
(c) 12 x 30 g = 360 g

(b) Container content = 300 mg/ml x 250 ml = 75000 mg = 75 g

Pack content: *(a) 6 x 75 g = 450 g,*
(b) 10 x 75 g = 750 g
(c) 12 x 75 g = 900 g

Step 2: Sum up the antimicrobial agent contained in all presentations and packages sold

Convert all contents of antimicrobial agent calculated under step 1 to the same weight unit and add up the total

Step 3: If necessary: convert the total sum of antimicrobial agent contained in all packages of all presentations sold to kg

Multiply the result from step 2 with an appropriate conversion factor to achieve the result in kg

2. (ii) – content of antimicrobial agent (chemical compound as declared on the product label) in International Units (IU) per weight, volume or other unit (for example millilitre, litre, kilogram, tablet) of content

Where the strength of the antimicrobial agent in the veterinary medicinal product is stated International Units (IU) per unit of finished product, an additional conversion step is necessary to obtain results in mg, g, or kg. Table 2 is used to convert content of antimicrobial agents declared in IU on the product label into mg for reporting to the OIE: either divide the total number of IUs of an antimicrobial agent by the value in the column 'International Units (IU) per mg' for this agent in table 2, or, if multiplication is preferred, multiply the total number of IUs with the conversion factor listed for the agent. To convert mg values into kg, please multiply the result of the conversion with 1 x 10^{-6} equalling 0.000001.

For some antimicrobial agents in veterinary medicinal products, the IU content or strength may be stated in respect to the active entity rather than to the chemical compound actually included; for example: a product may contain penethamate hydroiodide, or procaine benzylpenicillin, but the stated strength in IU refers to benzylpenicillin (product X containing penethamate hydroiodide, equivalent to xx IU benzylpenicillin, or, product Y containing procaine benzylpenicillin, equivalent to yy IU benzylpenicillin). For such cases, use the conversion factor for the relevant active entity listed in table 2 (in the examples used: benzylpenicillin). To convert mg values into kg, please multiply the result of the conversion with 1 x 10^{-6} equalling 0.000001.

If additional conversion factors are needed or have been used, please contact the OIE at antimicrobialuse@oie.int.

Step 1: Calculating the content of antimicrobial agent per package in IU

Multiply the amount of IU antimicrobial agent per unit of content with the total number of units contained in the package

$$Content\ of\ antimicrobial\ agent\ \ per\ package\ in\ IU$$
$$= Strength\ (amount\ IU\ antimicrobial\ agent\ per\ unit)\ x\ number\ of\ units\ per\ package$$

Step 2: Converting the content of antimicrobial agent per package in IU into mg

$$\text{Content of antimicrobial agent per package in } mg$$
$$= Content\ of\ antimicrobial\ agent\ \ in\ IU\ \text{x}\ conversion\ factor$$

Steps 3-4: Follow steps 2-3 described for (i)

Table 2: Conversion of International Units (IUs) of certain antimicrobial agents into mg and relevant active entities, based on the ESVAC conversion factors[1]

Antimicrobial agent in the veterinary medicine	Antimicrobial active entity for reporting to OIE	International Units per mg	Conversion factor to mg for multiplication
Bacitracin	Bacitracin	74	0.013514
Benzylpenicillin (penicillin G)	Benzylpenicillin	1666.67	0.0006
Chlortetracycline	Chlortetracycline	900	0.001111
Colistin methane sulfonate sodium (colistimethate sodium INN)	Colistin	12700	0.000079
Colistin sulfate	Colistin	20500	0.000049
Dihydrostreptomycin	Dihydrostreptomycin	820	0.00122
Erythromycin	Erythromycin	920	0.001087
Gentamicin	Gentamicin	620	0.001613
Kanamycin	Kanamycin	796	0.001256
Neomycin	Neomycin	755	0.001325
Neomycin B (Framycetin)	Neomycin B (Framycetin)	670	0.001492
Oxytetracycline	Oxytetracycline	870	0.001149
Paromomycin	Paromomycin	675	0.001481
Polymyxin B	Polymyxin B	8403	0.000119
Rifamycin	Rifamycin	887	0.001127
Spiramycin	Spiramycin	3200	0.000313
Streptomycin	Streptomycin	785	0.001274
Tobramycin	Tobramycin	875	0.001143
Tylosin	Tylosin	1000	0.001
Tetracycline	Tetracycline	950	0.001

2. (iii) – content of antimicrobial agent (chemical compound as declared on the product label) *in per cent (%) weight per weight (w/w) or weight per volume (w/v) of content*

The amount of antimicrobial agent contained in a veterinary medicine concerned may be stated in per cent weight per weight (% w/w) (example 1: product X contains tylosin 100% w/w or, example 2, product Y contains amoxicillin 22.2 % w/w) or in per cent weight per volume (% w/v) (example: product Z contains procaine benzylpenicillin 30% w/v). Such figures first need to be converted into mg/g, g/g, or mg/ml, followed by the calculations described under (i).

Converting % w/w: Conversion calculations are performed by relating the content of antimicrobial agent to 1 g of the finished product. Divide the percentage value by 100 to obtain the amount of antimicrobial agent in g per g finished product.

$$value\ antimicrobial\ agent\ in\ g\ per\ gram\ finished\ product = \frac{\frac{value\ (\%)}{100}\ x\ g}{1\ g\ (finished\ product)}$$

Example 1: Product X containing 100% w/w tylosin will contain 100/100 x g = 1 g tylosin per g finished product.

Example 2: Product Y containing 22.2% w/w amoxicillin will contain 22.2/100 = 0.222 g amoxicillin per g finished product.

Continue with Steps 1-3 of (i)

Converting % w/v: Conversion is based on the assumption that 1 ml of the products weighs 1000 mg. Multiply the percentage value with 10 to obtain the content in mg/ml.

$$value\ antimicrobial\ agent\ in\ g\ per\ ml\ finished\ product = \frac{value\ (\%) x\ 10\ \ x\ mg}{1\ ml\ (finished\ product)}$$

Example: Product Z containing 30% w/v benzylpenicillin will contain (30 x 10 x mg)/1ml, equal to 300 mg/ml benzylpencicillin.

Continue with Steps 1-3 of (i)

[1] http://www.ema.europa.eu/ema/pages/includes/document/open_document.jsp?webContentId=WC500189269

3. Additional recommendations for further conversions of quantities of antimicrobial agents

For pragmatic reasons the OIE accepts the reporting of antimicrobial agents in amounts of chemical compound as declared on the product label of the veterinary medicinal product. However, OIE Member Countries may wish to carry out further calculations to report amounts of active entity. If such further calculations are carried out, please describe them in the OIE template.

(i) Calculating the total amount expressed in weight of chemical compound as declared on the product label of a veterinary medicinal product into antimicrobial active entity (e.g. salt into base)

This step may be carried out once the steps described in section 1 or section 2. (i) have been completed.

As an example, for the antimicrobial agent tiamulin that is often available in the form of tiamulin hydrogen fumarate (the chemical compound as declared on the product label), the conversion formula to tiamulin (the active entity) would be:

Salt (including base): Tiamulin hydrogen fumarate MW 609.8
Base: Tiamulin MW 493.7
Conversion factor = MW base/MW salt (including base) = 0.81

Multiply the final result in kg obtained by following steps 1 to 3 with the appropriate conversion factor

$$Content\ of\ active\ entity\ (kg) = Content\ of\ chemical\ compound\ as\ listed\ on\ the\ label\ (kg)\ x\ \ conversion\ factor$$

(ii) The antimicrobial agent is in the form of a prodrug, expressed in weight

Where the antimicrobial agent contained in the veterinary medicinal product is a long-acting salt (example: benethamine benzylpenicillin) or a pro-drug (example: penethamate hydroiodide) and the content is stated in weight in reference to the actual chemical compound (example: product x contains 500 mg/ml benzylpenicillin benzathine), an additional conversion step as described below is needed to calculate the amount of active entity. When the antimicrobial agent is described in reference to the active entity (example: product y contains cloxacillin benzathine equivalent to 500 mg cloxacillin activity) the conversion using a prodrug conversion factor described below is not necessary.

Taking the prodrug conversion factors used by the European Surveillance of Veterinary Antimicrobial Consumption (ESVAC) program managed by the European Medicines Agency, as a starting point, table 3 lists the suggested conversion factors for relevant long-acting salts and prodrugs. The amount of the actual chemical compound as declared on the product label (example: benzylpenicillin benzathine) needs to be multiplied with the prodrug conversion factor to obtain the corresponding amount of the active entity (example: benzylpenicillin.

If additional conversion factors are needed or have been used, please contact the OIE at antimicrobialuse@oie.int.

Table 3: Conversion of content stated in mg, g or kg of long-acting salts and prodrugs of antimicrobial agents in the veterinary product into corresponding mg, g or kg antimicrobial active entity for reporting to the OIE, based on the ESVAC conversion factors[2]

[2] http://www.ema.europa.eu/ema/pages/includes/document/open_document.jsp?webContentId=WC500189269

Antimicrobial agent (prodrug)	Active entity	Prodrug conversion factor for multiplication
Benethamine benzylpenicillin	Benzylpenicillin	0.65
Benzathine benzylpenicillin	Benzylpenicillin	0.74
Cefapirin benzathine	Cefapirin	0.41
Cefalexin benzathine	Cefalexin	0.36
Cloxacillin benzathine	Cloxacillin	0.43
Oxacillin benzathine	Oxacillin	0.69
Penethamate hydroiodide	Benzylpenicillin	0.63
Procaine benzylpenicillin	Benzylpenicillin	0.61

Step 1–3: As described in section 2. (i)

Step 4: Multiply the final result in kg obtained by following steps 1 to 3 with the appropriate conversion factor listed in table 3

$$Antimicrobial\ agent\ (active\ entity)(kg) = antimicrobial\ agent\ (chemical\ compound\ as\ declared\ on\ the\ product\ label)(kg)\ x\ prodrug\ conversion\ factor$$

For bulk quantities of antimicrobial agents in form of prodrugs, the additional step 2 described below should be applied after the calculations described in section 1.

Step 2: If the antimicrobial agent is a long-acting salt or prodrug listed in table 3 above, additionally multiply with the corresponding conversion factor.

$$Antimicrobial\ agent\ (active\ entity)(kg) = Step\ 1\ antimicrobial\ agent\ (chemical\ compound\ as\ declared\ on\ the\ product\ label)\ kg\ x\ prodrug\ conversion\ factor$$
