# Supplementary material for: Determination of antimicrobial use in commercial poultry farms in Plateau and Oyo States, Nigeria
Source: Antimicrob Resist Infect Control. 2023 Apr 10;12:30. doi: 10.1186/s13756-023-01235-x (PMC10084607; doi:10.1186/s13756-023-01235-x)
Supplement: Supplementary file 2 — Additional file 2. List of antibiotics used on farms (Plateau). [file 13756_2023_1235_MOESM2_ESM.docx]

| **List of antibiotics used on farms (Plateau)** | | |
| --- | --- | --- |
| **S/N** | **Antibiotic** | **Frequency** |
| 1 | Doxygen (Doxycycline and Gentamycin) | 8 |
| 2 | Enrofloxacin | 3 |
| 3 | Amoxycol (Amoxicillin 200mg, Colistin sulphate 1,000,000 IU) 1 Litre | 5 |
| 4 | Amoxicolinor (Amoxicillin 200mg, Colistin sulphate 1,000,000 IU) | 1 |
| 5 | Penicillin | 1 |
| 6 | Biofloxacin (Enrofloxacin) | 1 |
| 7 | Oxytetracycline | 4 |
| 8 | N.C.O. Mix (Florfenicol (150mg), Neomycin sulphate (180mg), Colistin sulphate (1,200,000 IU)) | 1 |
| 9 | Oxyfuravet | 1 |
| 10 | Neodox (Neomycin and Doxycycline) | 1 |
| 11 | Neodoxinor (Neomycin 200mg, Doxycycline 200mg) | 1 |
| 12 | Kenflox | 2 |
| 13 | Conflox | 1 |
| 14 | Ciprofloxacin (200mg) | 4 |
| 15 | Ciprosol-200 (Ciprofloxacin 200mg) | 1 |
| 16 | Centre -Cipro (200mg) 1 Litre | 1 |
| 17 | Biodox (Doxycycline 100mg plus Lactobacillus 5Billion spores) Capsules | 1 |
| 18 | Gentamycin | 1 |
| 19 | Neodox (Neomycin nd Doxycycline) | 1 |
| 20 | Neooxyceryl | 1 |
| 21 | Gentylo (Gentamycin and Tylosin) | 1 |
| 22 | Colistin | 3 |
| 23 | Ceryl treat - Zogceryl (Oxytet 5,000mg, Colistin 2,500mg, Neomycin 200mg) plus vitamins | 2 |
| 24 | Floricol (Florfenicol 100mg) 100ml | 6 |
| 25 | Keproceryl | 2 |
| 26 | Furatadol | 1 |
| 27 | Neofuraseryl (Neomycin 100mg, oxytetracycline 50,000mg, colistin sulfate 30,000,000 IU plus vitamins) | 2 |
| 28 | Streptomycin | 1 |
| 29 | Robioxacin 200 (Enroflox 20%) | 1 |
| 30 | Agentadox 10/5 (gentamycin 100mg, Doxycycline 50mg) |  |
| 31 | Nemovit | 1 |
| 32 | Florum 20% 1000ml | 1 |
| 33 | Thiamphenicol) 25% 1L | 1 |
| 34 | Flumcol | 1 |
| 35 | Floxinor (Enrofloxacin 20%) | 1 |
| 36 | Neo-furamycine plus ( Furazolidone 6000mg, Neomycin 200mg, Oxytetreacycline 500mg, Streptomycine 200mg, Erythromycine 3500mg, Chloramphenicol 2000mg) |  |
| 37 | Enrocare (Enrofloxacin) | 1 |
| **Human health preparations** | | |
| 38 | Septrin (Sulphur/Trimethoprim) |  |
| 39 | Gentamicin injection (80mg, 2ml ampule | 1 |
|  | Subtotal | 39 |
