# Supplementary material for: Determination of antimicrobial use in commercial poultry farms in Plateau and Oyo States, Nigeria
Source: Antimicrob Resist Infect Control. 2023 Apr 10;12:30. doi: 10.1186/s13756-023-01235-x (PMC10084607; doi:10.1186/s13756-023-01235-x)
Supplement: Supplementary file 3 — Additional file 3. List of antibiotics used on farms (Oyo state). [file 13756_2023_1235_MOESM3_ESM.docx]

| **List of antibiotics used on farms (Oyo state)** | | |
| --- | --- | --- |
| **S/N** | **Antibiotic** | **Frequency** |
| 1 | Doxygen (Doxycycline and Gentamycin) | 7 |
| 2 | Enrocare (Enrofloxacin) | 13 |
| 3 | Embaceryl (Tylosin 65mg/colistin 300,000 IU) | 2 |
| 4 | Keproceryl | 4 |
| 5 | Amoxicol | 9 |
| 6 | Centre Gentamicin Tylosin (gentamicin 12%, tylosin 20%) | 1 |
| 7 | Centre Tylo | 1 |
| 8 | Coligent (colistin 25,000IU, gentamicin 50mg) | 1 |
| 9 | Colistin sulphate | 2 |
| 10 | Conflox | 1 |
| 11 | Floxinor 20% | 2 |
| 12 | Furataldone | 1 |
| 13 | Genta tylo | 4 |
| 14 | Moosun tylosin Doxycycline ( Tylosin 20g/Doxycycline 20g) | 1 |
| 15 | N.C.O. | 4 |
| 16 | Neoceryl | 11 |
| 17 | Nemovit | 1 |
| 18 | Oxytetracycline 20% | 1 |
| 19 | Toltrazurilceryl | 1 |
| 20 | Tylodox | 7 |
| 21 | Tylosin | 3 |
| 22 | Agentadox (100 mg gentamycin sulphate and 50 mg doxycycline hyclate). | 3 |
| 23 | Embaceryl(Tylosin base 3,800mg, Oxytetracycline 4,000mg, Neomycin sulphate 1,200mg, Colistin sulphate 30,000,000 IU) | 3 |
| 24 | Maxiceryl (colistin 225000iu/ oxytet 5000mg/ neomycin 5000mg) | 1 |
